# Application of 5G Technology to Conduct Real-Time Teleretinal Laser Photocoagulation for the Treatment of Diabetic Retinopathy

**DOI:** 10.1001/jamaophthalmol.2021.2312

**Published:** 2021-07-08

**Authors:** Huan Chen, Xuefeng Pan, Jingyuan Yang, Jing Fan, Mingwei Qin, Hu Sun, Jinxin Liu, Na Li, Daniel Shu Wei Ting, Youxin Chen

**Affiliations:** 1 Department of Ophthalmology, Peking Union Medical College Hospital, Key Laboratory of Ocular Fundus Diseases, Chinese Academy of Medical Sciences and Peking Union Medical College, Beijing, China; 2 Department of Ophthalmology, Huzhou First People’s Hospital, Huzhou, China; 3 Department of Telemedicine Center, Peking Union Medical College Hospital, Chinese Academy of Medical Sciences and Peking Union Medical College, Beijing, China; 4 Clin Medical Instrument Co, Ltd, Shanghai, China; 5 China Mobile Communications Group Co, Ltd, Beijing, China; 6 Singapore National Eye Center, Duke-NUS Medical School, Singapore

## Abstract

**Importance:**

Interest in teleophthalmology has been growing, especially during the COVID-19 pandemic. The advent of fifth-generation (5G) wireless systems has the potential to revolutionize teleophthalmology, but these systems have not previously been leveraged to conduct therapeutic telemedicine in the ophthalmology field.

**Objective:**

To assess the feasibility of 5G real-time laser photocoagulation as a telemedicine-based treatment for diabetic retinopathy (DR).

**Design, Setting, and Participants:**

This was a prospective study involving a retinal specialist from the Peking Union Medical College Hospital in Beijing, China, who performed online 5G real-time navigated retinal laser photocoagulation to treat participants with proliferative or severe nonproliferative DR who had been recruited in the Huzhou First People’s Hospital in Zhejiang Province, China, located 1200 km from Beijing from October 2019 to July 2020.

**Interventions:**

These teleretinal DR and laser management procedures were conducted using a teleophthalmology platform that used the videoconference platform for teleconsultation, after which telelaser planning and intervention were conducted with a laser system and a platform for remote computer control, which were connected via 5G networks.

**Main Outcomes and Measures:**

Diabetic eye prognosis and the real-time laser therapy transmission speed were evaluated.

**Results:**

A total of 6 participants (9 eyes) were included. Six eyes were treated via panretinal photocoagulation alone, while 1 eye underwent focal/grid photocoagulation and 2 eyes underwent both panretinal photocoagulation and focal/grid photocoagulation. The mean (SD) age was 53.7 (13.6) years (range, 32-67 years). The mean (SD) duration of diabetes was 14.3 (6.4) years (range, 3-20 years). The mean (SD) logMAR at baseline was 0.32 (0.20) (20/30 Snellen equivalent). Retinal telephotocoagulation operations were performed on all eyes without any noticeable delay during treatment. The mean (SD) number of panretinal photocoagulation laser spots per eye in 1 session was 913 (243).

**Conclusions and Relevance:**

This study introduces a novel teleophthalmology paradigm to treat DR at a distance. Applying novel technologies may continue to ensure that remote patients with DR and other conditions have access to essential health care. Further studies will be needed to compare this approach with the current standard of care to determine whether visual acuity or safety outcomes differ.

## Key Points

**Question:**

How feasible is fifth-generation (5G) real-time telemedicine-mediated laser photocoagulation as a treatment for diabetic retinopathy?

**Findings:**

In this study, a retinal specialist in Beijing, China, performed an online 5G real-time navigated retinal laser photocoagulation procedure on 6 participants (9 eyes) with diabetic retinopathy located in Huzhou, China. All procedures were able to be completed without noticeable delay, and no safety issues were identified.

**Meaning:**

The combination of a 5G high-speed network and navigated retinal laser photocoagulation may enable a novel teleophthalmology paradigm that can provide essential remote health care to patients with diabetic retinopathy.

## Teaser

This study assesses the feasibility of real-time telephotocoagulation treatment for diabetic retinopathy.

## Introduction

Telemedicine entails the remote provision of health care services using telecommunication devices including smartphones and other wireless devices with remote video connections. Telemedicine is transforming the delivery of health care to millions of people who are unable to visit hospitals and clinics. Owing to the unique structural properties of the eye, which enable clinicians to directly visualize the neural tissue, connective tissue, vasculature, and any retinal or choroidal lesions, ophthalmology has been at the forefront of practical advances in telemedicine.^1^

The reliability of teleophthalmology-based evaluation and screening approaches have been demonstrated in multiple studies to date. Several programs focused on the treatment of diabetic retinopathy (DR) have successfully implemented teleophthalmology approaches. In a 2015 meta-analysis of 20 studies, the overall diagnostic sensitivity and specificity of telemedicine-based DR screening was more than 70% and 90%, respectively.^2^ However, surprisingly few telemedicine-based approaches to therapeutic treatment for patients with DR have been reported to date.^3^ Kozak et al^3^ reported the successful retinal telephotocoagulation treatment of 16 eyes with DR after transferring retinal images and fluorescein angiograms captured in a clinic in Saudi Arabia to another clinic in the US, with treatment being conducted in Saudi Arabia according to the offline treatment plans drafted by ophthalmologists in the US. The laser device used in this study was able to conduct automated retinal photocoagulation after treatment plan input. This novel asynchronous telemedicine strategy highlights the promise of therapeutic telemedicine in the field of DR.^3^

Telephotocoagulation treatment with real-time online monitoring techniques is a much safer medical approach than offline telemedicine. The development of fifth-generation (5G) wireless systems has the potential to revolutionize telemedicine as a means of treating DR,^4^ making it feasible to deliver real-time health care–related services from a distance to remote patients. During the COVID-19 pandemic, the ability to conduct telemedicine in collaboration with local ophthalmologists to monitor and treat DR has become increasingly crucial. However, few studies have explored the feasibility of real-time telephotocoagulation treatment, which may meet a critical unmet public health need in the context of the rising prevalence of DR.

The use of 5G technology to communicate directly with physicians and to share medical records through an online platform has the potential to improve the quality of telemedicine-based DR treatment while engendering trust and enhancing patient-physician relationships. The development of laser photocoagulation instruments and new telecommunication platforms have highlighted the promise of therapeutic telemedicine as a means of treating DR. Without access to such treatment, many patients in rural areas will not be able to benefit from high-quality treatments. This is particularly true in China, owing to a shortage of retinal specialists and an imperfect training system such that most comprehensive ophthalmologists in rural China do not know how to perform retinal laser treatment properly and can only transfer patients with DR to first- or second-tier cities for further care. As such, we herein conducted teleophthalmology interventions for patients with DR using a 5G-based teleconsultation platform and a real-time automated retinal laser device. The primary goal of this study was to assess the feasibility of real-time telephotocoagulation treatment for DR. The secondary goal of this study was to investigate a potential health care solution for patients under adverse circumstances, as in the context of the COVID-19 pandemic, which has limited the ability of patients to travel for health care.

## Methods

This was a double-site, nonrandomized, prospective study performed between Peking Union Medical College Hospital (PUMCH) in Beijing, China, and Huzhou First People’s Hospital in Huzhou, China (eMethods and eFigure 1 in the Supplement). Institutional review board approvals were obtained from both PUMCH and Huzhou First People’s Hospital. All study participants provided written informed consent specific for 5G real-time telemedicine-mediated laser photocoagulation, and all evaluations and laser treatments in this study were free for participants as compensation. The study was conducted in accordance with the tenets of the Declaration of Helsinki.^5^

### Participants

Six participants from Huzhou (≥18 years of age) presenting with severe proliferative DR (PDR) or nonproliferative DR who were eligible to undergo retinal laser photocoagulation were enrolled in this study. Participants were excluded if they exhibited media opacities that would disrupt laser treatment, including intravitreal hemorrhage or severe cataracts. All participants underwent comprehensive ophthalmic examinations at a local hospital, including visual acuity testing, noncontact intraocular pressure measurements, slitlamp biomicroscopy, dilated ophthalmoscopic examinations, color fundus photography, and fluorescein angiography prior to laser treatment.

### Telemedicine Platform Setting for Real-Time 5G Telephotocoagulation

**Figure 1.**
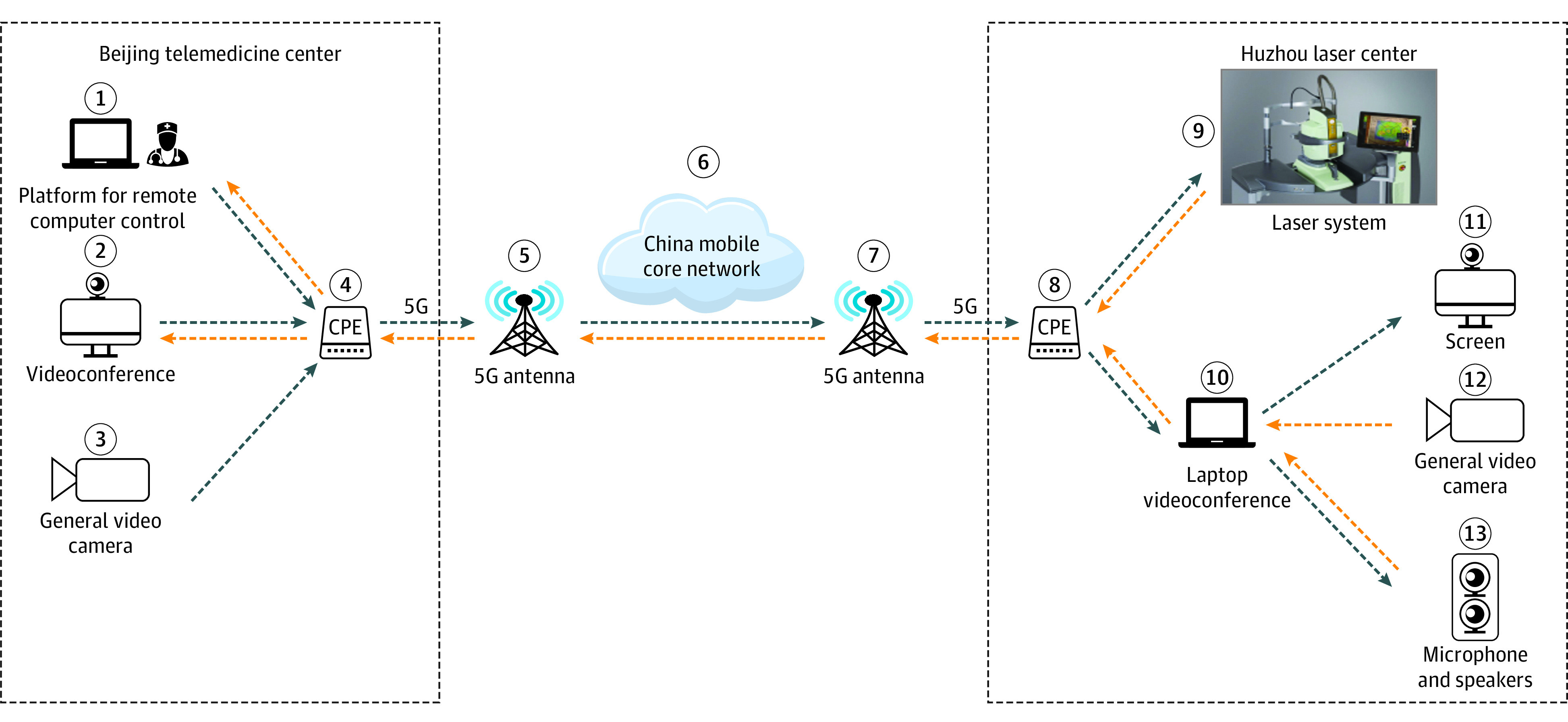
Equipment and Network Configuration for Real-Time Telephotocoagulation 1, A computer on which a platform for remote computer control had been installed was located in the Peking Union Medical College Hospital (PUMCH) telemedicine center in Beijing, China, and was connected to the fifth-generation (5G) customer premises equipment (CPE) using an ethernet cable (4). The device received an image from the screen of the laser system (9). The retinal specialist was then able to remotely draw the laser treatment plan on this screen, and the resultant plant was transmitted back to the laser system (9) at the laser center in Huzhou, China. 2, This video platform was located in the PUMCH telemedicine center and was connected to the CPE via ethernet cable (4). This platform provided a general overview of the Huzhou laser center (12) and the laser system screen (9), while also receiving the audio (13) from the laptop equipped with videoconferencing (10) in Huzhou. This device also transmitted a general view (3) and the audio from the PUMCH telemedicine center to Huzhou. 3, A general view of the PUMCH telemedicine center was recorded with a video camera. (4) The CPE was provided by mobile operators, and image streams and audio were sent to the Huzhou laser center through this device. (5) The CPE device received a 5G signal from an antenna located in PUMCH. 6, This core network provided an independent and dedicated high-priority transmission strategy and an exclusive backup for 5G network transmission. 7, The CPE devices received a 5G signal from an antenna located in Huzhou First People’s Hospital in Huzhou, China. 8, The CPE in Huzhou was placed in the laser center and was connected to both the laptop (10) and the laser system (9) in this center. 9, This laser system was located in the Huzhou laser system and was connected to the CPE via ethernet. It received images from the laptop in PUMCH (1) via 5G transmission. 10, Videoconference software was installed on this laptop, which received a general view (12) and audio (13) inputs and was connected to the screen (11) in Huzhou using an HDMI cable. 11, The Huzhou First People’s Hospital provided a 27-inch screen that was located in the laser center and projected both the general view and the laser plan from the PUMCH telemedicine center. 12, A general overview of the laser center was recorded with a video camera, enabling the retinal specialist to view what was occurring in the laser center. 13, The retinal specialist’s voice was transmitted to the laptop (10) and speakers.

The telemedicine platform used for real-time 5G telephotocoagulation in this study (Figure 1) was composed of 4 parts: (1) a laser system (Navilas; OD-OS GmbH) for navigated retinal photocoagulation; (2) the TeamViewer platform (TeamViewer GmbH) for remote computer control; (3) videoconference software (Cisco Systems, Inc) for teleconsultation; and (4) 5G networks (China Mobile Communications Group Co, Ltd) for high-speed data transmission.

### Teleophthalmology Intervention

The teleophthalmology interventions described herein were conducted on October 27, 2019, and December 15, 2019 (eFigure 2 in the Supplement). Prior to initiating these interventions, an experienced retinal specialist (Y.C.) received and reviewed the abovementioned medical records for each patient that had been transmitted using the telemedicine platform. The retina specialist then discussed conditions and treatment plans with the patient and local ophthalmologists via videoconferencing. After providing a detailed explanation of the benefits and risks of 5G telemedicine-mediated retinal laser photocoagulation, participants were informed of the importance of this treatment and provided written informed consent. Videoconferencing plays an important role in improving ocular outcomes for these participants, and it is important to note that teleophthalmology is not inferior to in-person care because treatment planning is conducted largely based on imaging data.

**Figure 2.**
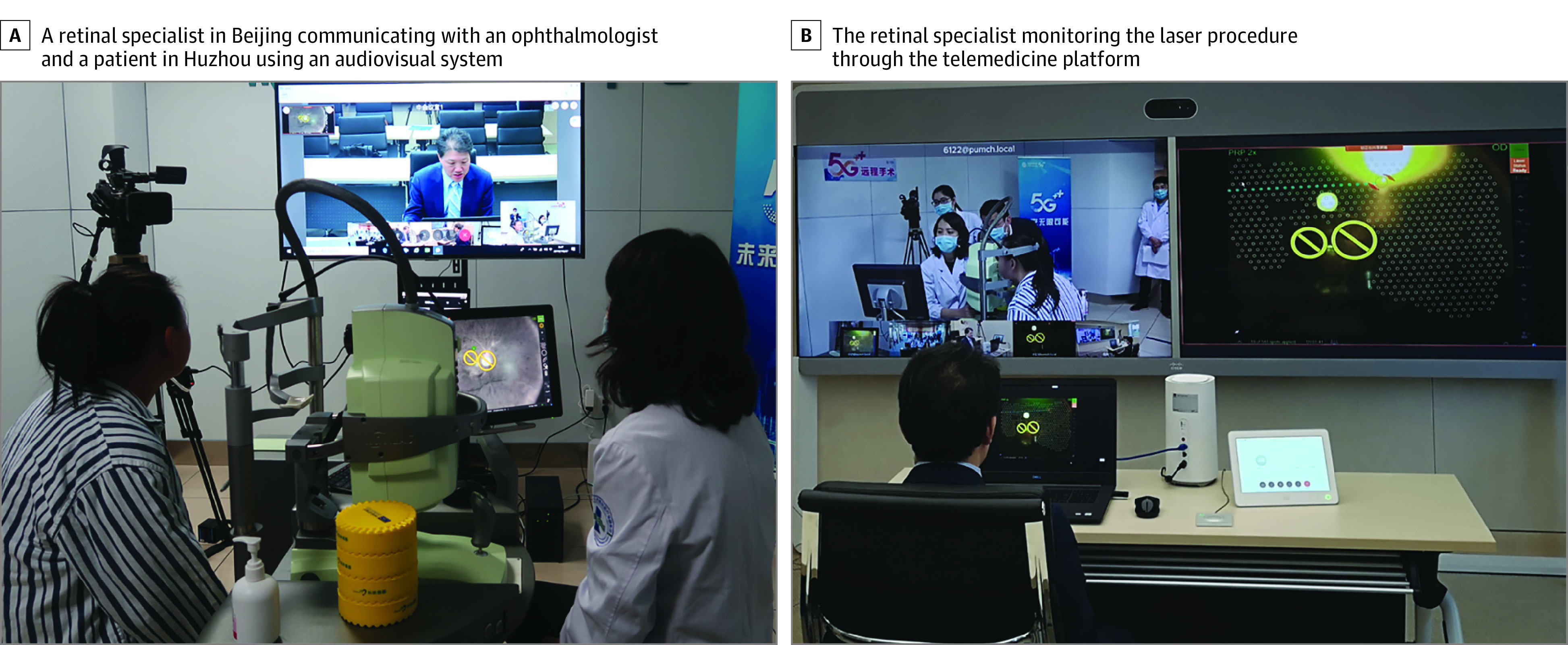
5G-Assisted Real-Time Teleretinal Laser Photocoagulation Treatment Scenario For the majority of the telephotocoagulation procedure, the retinal specialist in Beijing, China, remained in communication with a local ophthalmologist and the patient in Huzhou, China, using an audiovisual system, with the retinal specialist drawing treatment plan on the screen of a laptop that was visible to the ophthalmologist in Huzhou on the laser machine display (A). Then the retinal specialist in Beijing was able to monitor the entire laser procedure through the telemedicine platform via fifth-generation (5G) network (B).

An online-navigated retinal laser photocoagulation operation was conducted for each patient. The laser system was initially designed to improve automatic retinal laser application via the introduction of navigation functions, allowing for fast and secure retinal photocoagulation of the designated areas using a fluorescein angiography or a color fundus photograph taken with the system. We incorporated additional real-time remote control functions into this navigated laser system as a part of our telemedicine platform as follows. The retinal specialist in Beijing remotely controlled the laser device in Huzhou using a computer connected to the platform for remote computer control. The operation interface and real-time fundus video, as well as a simultaneous live video from 2 cameras (with one providing a panoramic view of the laser treatment room and another providing close-up shots of the digital laser device monitor) were transmitted from Huzhou to the retinal specialist in Beijing via videoconferencing. The retinal specialist planned individual patient treatment approaches by swiping with his finger to define laser areas and caution zones (nonlaser areas) on a color fundus photograph presented on the telemedicine platform screen, after which the laser system performed photocoagulation automatically based on this treatment plan. Throughout this process, the retinal specialist monitored the laser effect via videoconferencing to ensure safety and real-time synchronization (Figure 2). Other laser parameters were also defined by the specialist in Beijing before treatment or were adjusted intraoperatively (Video). After topical anesthesia, a sterilized ocular lens (Ocular Mainster PRP lens; Ocular Instruments, Inc) was used for the process of laser treatment. All data mentioned above were transmitted via 5G networks.

**Video.** Use of 5G Technology For Real-Time Telemedicine-Mediated Laser Photocoagulation Treatment of Diabetic Retinopathy A retinal specialist in Beijing, China, communicates with a local ophthalmologist and the patient in Huzhou, China, using an audiovisual system, with the retinal specialist defining areas for laser treatment and caution zones (nonlaser areas) on a color fundus photograph. The treatment plan is visible to the ophthalmologist in Huzhou on the laser machine display. The laser system performs photocoagulation automatically, with the retinal specialist in Beijing monitoring the entire procedure through the telemedicine platform via 5G network.

### Laser Application Quality Control and Evaluation

The 5G network rate and network latency were recorded as a means of evaluating the telemedicine platform transmission speed. During the laser procedure, a color fundus photograph was constantly captured to record the condition of the fundus. These images were evaluated by a single retina specialist (Y.C.) who assessed the teleophthalmology intervention in these participants. Laser spot energy and exposure times were adjusted in real time adjusted based on the instructions of the retina specialist.

### Statistical Analysis

Data were given as means and SDs. No adjustment was made to the 2-sided *P* values for multiple analyses. *P *values were statistically significant at less than .05. SPSS statistical software version 22.0 (IBM Corp) was used for all statistical testing.

## Results

### Clinical Outcomes

In total, 3 participants (4 eyes) with severe nonproliferative DR and 3 participants (5 eyes) with PDR were enrolled in this study, with a mean (SD) age of 53.7 (13.6) years (range, 32-67 years) and a mean (SD) diabetes duration of 14.3 (6.4) years (range, 3-20 years). The mean (SD) logMAR at baseline was 0.32 (0.20) (Table).

**Table.** Baseline Demographics, Visual Acuity, Laser Pattern, and Anatomic Outcomes of Individuals Treated With Telephotocoagulation

Participant	Age in decades, y	Duration of diabetes, y	Eye	DR stage	Treatment plan	Laser spots	BCVA				CST, μm		Follow-up duration, mo
							Baseline		Follow-up		Baseline	Follow-up	
							Snellen	LogMAR	Snellen	LogMAR			
1	30s	3	Right	High-risk PDR	PRP	810	20/50	0.4	20/60	0.48	467	436	1
			Left	High-risk PDR	PRP	947	20/30	0.18	20/40	0.3	331	295	1
2	40s	12	Left	Non–high-risk PDR	PRP	1133	20/30	0.18	20/40	0.3	197	201	1
3	60s	14	Right	Severe non-PDR	PRP	801	20/25	0.1	20/25	0.1	263	257	1
			Left	Severe non-PDR	PRP	558	20/25	0.1	20/25	0.1	272	275	1
4	50s	17	Right	Non–high-risk PDR	PRP + focal/grid	969 + 331	20/40	0.3	20/50	0.4	322	341	1
			Left	High-risk PDR	PRP + focal/grid	1139 + 307	20/50	0.4	20/60	0.48	435	428	1
4^a^	50s	17	Right	Non–high-risk PDR	PRP	684	20/50	0.4	20/50	0.4^b^	341	249^b^	6
			Left	High-risk PDR	PRP	739	20/60	0.48	20/60	0.48^b^	428	301^b^	6
5	60s	20	Right	Severe non-PDR	PRP	1353	20/60	0.48	20/200	1.0^b^	296	577^b^	6
6	60s	20	Right	Severe non-PDR	Focal/grid	169b	20/100	0.7	20/200	1.0^b^	403	450^b^	6

Abbreviations: BCVA, best-corrected vision acuity; CST, central subfield thickness; DR, diabetic retinopathy; PDR, proliferative diabetic retinopathy; PRP, panretinal photocoagulation.

^a^ Patient 4 received the first laser treatment on October 27, 2020, and received PRP top-up treatment after a comprehensive teleconsultation by retinal specialist in Beijing on December 15, 2020.

^b^ Owing to the outbreaks of COVID-19, the follow-up data were collected 6 months later.

### Retinal Laser Photocoagulation Outcomes

Six eyes underwent panretinal photocoagulation (PRP), 1 eye underwent focal/grid photocoagulation, and 2 eyes underwent both PRP and focal/grid photocoagulation. The mean (SD) number of PRP laser spots per eye in 1 session was 913 (243) (range, 558-1353), the mean (SD) power used was 198 (45) mW (range, 95-258 mW), and the mean (SD) laser duration was 107 (15) milliseconds (range, 95-150 milliseconds). All treatment plans were accomplished successfully, and there were no adverse events during photocoagulation.

**Figure 3.**
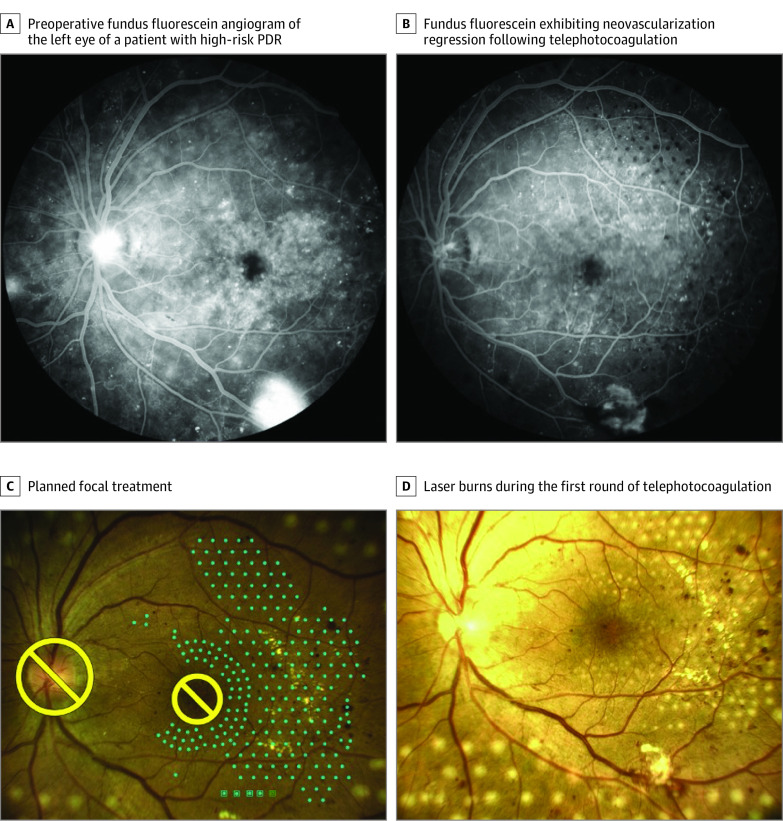
Patient With PDR Who Received 5G-Assisted Real-Time Teleretinal Laser Photocoagulation A, Preoperative fundus fluorescein angiogram of the left eye from a patient with high-risk proliferative diabetic retinopathy (PDR) exhibiting neovascularization leakage at the disk and additional neovascularization in an area greater than a half disc in size. B, Fundus fluorescein angiogram exhibiting neovascularization regression following telephotocoagulation. C, Image of the planned focal treatment (blue dots). D, Laser burns during the first round of telephotocoagulation.

**Figure 4.**
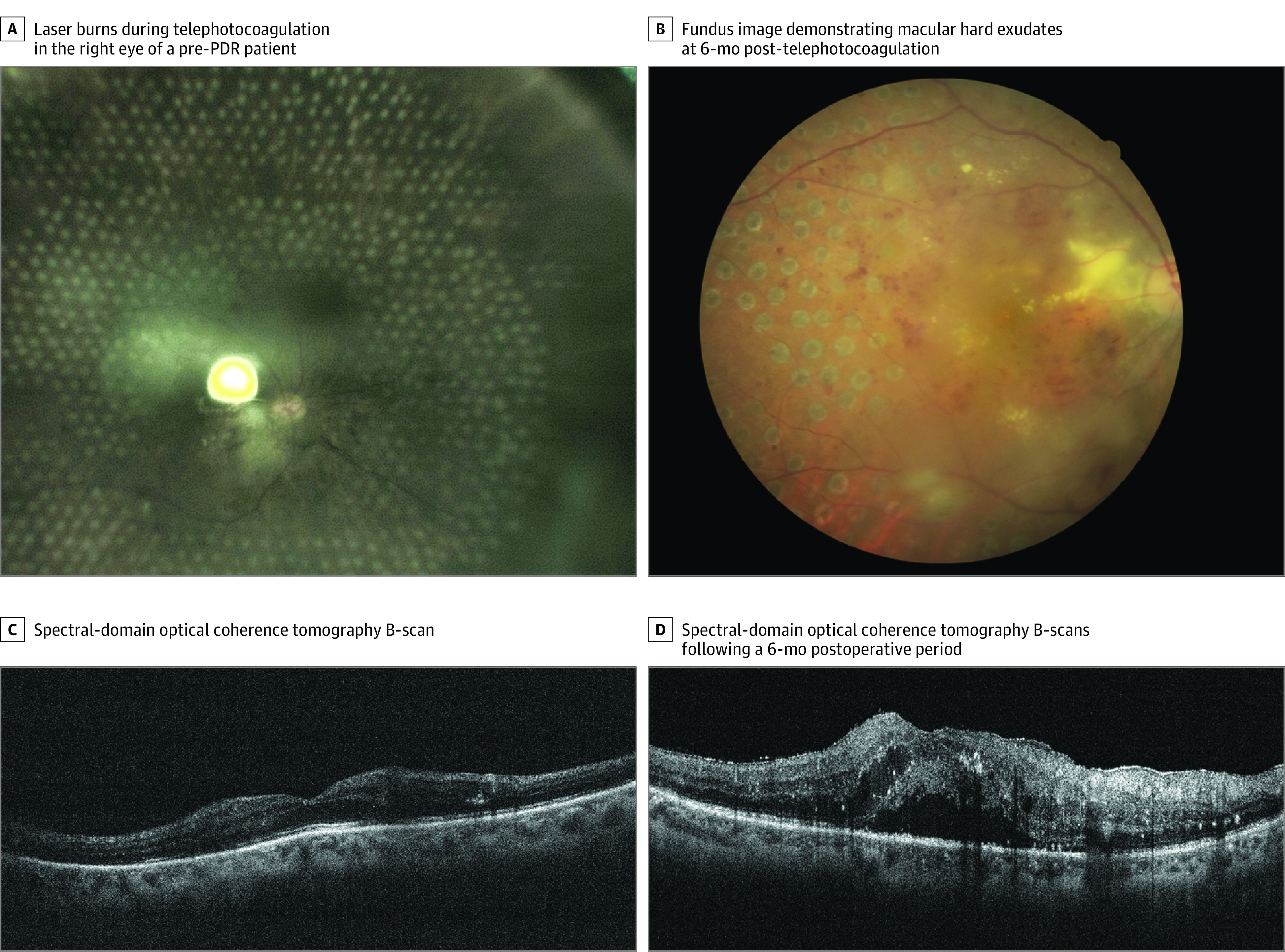
Patient With Pre-PDR Who Received 5G-Assisted Real-Time Teleretinal Laser Photocoagulation Who Did Not Follow Up in Time During the COVID-19 Pandemic A, Image of laser burns during telephotocoagulation in the right eye of a patient with pre–proliferative diabetic retinopathy (PDR). B, Fundus image demonstrating macular hard exudates at 6 months after telephotocoagulation. C, Spectral-domain optical coherence tomography B-scan revealing hard exudates but no apparent macular edema prior to telephotocoagulation. D, Spectral-domain optical coherence tomography B-scans were repeated following a 6-month postoperative period during which the patient had poor glucose control and underwent cataract surgery, revealing worsening macular edema and hard exudates.

Four participants (7 eyes) experienced no significant changes in visual acuity throughout a 1-month follow-up period (Figure 3). Owing to the COVID-19 pandemic, 2 participants (2 eyes) did not undergo follow-up until 6 months postoperatively, at which time they reported decreases in visual acuity and poor glucose control, with 1 patient having undergone cataract surgery in a local hospital (Figure 4). Two eyes in 1 patient necessitated top-up PRP owing to the presence of persistent neovascularization 1 month after the first teleretinal laser photocoagulation procedure. No participants required additional vitreoretinal surgery owing to vitreous hemorrhage or the development of neovascularization.

### Telemedicine Platform Outcomes

All medical records were sent from Huzhou to Beijing for treatment planning, and all treatment plans were successfully transmitted without any errors. The mean (SD) data upload and download speeds were 88.45 (5.39) MB/s and 853.63 (52.71) MB/s, respectively, with no loss of signal quality. The mean (SD) network latency time was 20 (3) milliseconds. No significant signal loss was observed, nor was there any evidence of image buffering or pixelation. No complications occurred during photocoagulation or any other stage of this procedure.

The mean (SD) video call time was 23.4 (5.6) minutes, including 3.5 (1.1) minutes for treatment planning and 18.1 (5.2) minutes per eye for automatic laser application.

## Discussion

The advent of new technologies and devices that can be used to remotely manage certain diseases has led to telemedicine emerging as a valuable tool for providing quality care to patients at a distance. Owing to technical constraints, asynchronous teleophthalmology is more common than real-time teleophthalmology. Combining asynchronous and real-time tele-examination approaches has proven to be effective in the field of ophthalmology.^1,6,7^ However, real-time telemedicine-based approaches to directly treating ophthalmic patients have not been implemented to date. Herein, we described a telemedicine-based treatment for DR, and we evaluated and confirmed the feasibility and potential safety of such remote retinal laser photocoagulation, although only among 6 participants.

Herein, we used an integrated multifunctional telemedicine platform that was able to facilitate medical record sharing, remote device operation, monitoring, and video calling in a real-time format using 5G networks such that ophthalmologists 1000 km apart were able to cooperatively provide patient care without any perceivable time delay.^8,9^ Visual guidance typically initiates within 200 to 300 milliseconds of observing a visual stimulus,^10^ and we detected a mean network latency of just 20 milliseconds in the present study. Such a short delay should have no meaningful effect on decision-making.

The development of digital automated laser devices and 5G networks has made real-time telephotocoagulation for the treatment of fundus diseases an ideal telemedicine strategy. In contrast with many other disease types that require physical evaluation to develop an appropriate treatment plan, laser photocoagulation treatment for DR is primarily dependent on multimedia data derived from imaging studies, which can be readily transmitted through high-speed 5G networks.

In March 2019, a Chinese neurosurgeon used 5G networks to remotely place a deep brain stimulation implant in a patient with Parkinson disease located approximately 3000 km away. Many other 5G-based remote laparoscopic and urological operations have been conducted since that time.^8,9^ However, there are certain limitations to telemedicine, such that many of these operations were in fact telementored surgeries with a range of practical limitations. For example, less experienced surgeons may have difficulties understanding the instructions of the remote surgeon, who would be unable to take over in the event of a complication. Additionally, these operations cannot be stopped until they are completed, and an experienced local surgeon must therefore remain on standby to resolve any potential issues. In contrast, telephotocoagulation procedures do not cause wounding and can be terminated at any stage of the procedure, making them more practical and safe to implement in the context of 5G-assisted real-time teletreatment.

As 5G networks offer a stable high-speed signal, they also offer an opportunity to overcome clinical barriers to the quality of patient care, evaluation, and relationships with physicians. Remote telemedicine can often lead to reduced quality patient-physician interactions, while video consultation and real-time monitoring can help to engender trust and familiarity between patients and physicians with whom they lack an established relationship.^11,12,13^

It is important to note that not all patients achieved satisfactory short-term outcomes in this study, potentially owing to the inherent limitations of navigated laser treatment.^14^ These patients with diabetes included individuals living in rural areas who were unable to undergo standard treatments for systemic diseases or to regularly take anti–vascular endothelial growth factor agents or other drugs. The underdeveloped regions where these individuals live are ideal targets for telemedicine development. However, owing to restrictions associated with local economic factors and national medical insurance, most rural Chinese patients are unable to access regular comprehensive treatments that are in accordance with current practice guidelines. As such, multiple medical disciplines must cooperate with one another to facilitate comprehensive remote telemedicine for these patients. The present study represents an important step in this direction for the ophthalmology field, as we were able to successfully implement a continuous remote laser treatment in some patients, and no patients included herein experienced any deterioration with respect to DR stage or classification. However, it should be noted that 15% of eyes with PDR treated with PRP require vitrectomy within 2 years,^15^ so continued close follow-up after PRP is also important. Virtual vision self-checks may be of help in the future.

Telemedicine development should be focused on areas subjected to the abovementioned limitations. In developed areas, teleophthalmology primarily focuses on achieving better visual outcomes, whereas in underdeveloped areas it primarily seeks to prevent blindness. The telemedicine approaches described herein offer new opportunities for preventing progressive DR and blindness in patients with diabetes. As such, patients will experience a greater economic burden if they develop PDR, and the design of telemedical models suitable to treat these patients is essential.

While the telemedicine approach described herein is feasible, telemedicine-based treatment also inevitably results in health care fragmentation, which has the potential to result in conflicting recommendations from disconnected physicians. To reduce the risk of this outcome, tripartite video consultation between experienced physicians, local physicians, and patients will ensure accurate diagnoses and facilitate uniform acceptance of and adherence to an appropriate treatment plan.^16^

The novel technologies used in the present study can be leveraged to educate local ophthalmologists regarding the retinal laser photocoagulation procedure, in addition to improving patient access to services and lowering associated health care costs.^17,18^ As the international burden of chronic diseases continues to rise and the distribution of physicians remains insufficient to combat this issue, video calling–based telemedicine represents a vital avenue that can improve access to education.^19^ Using imaging devices and high-speed network transmission to visualize lesions in real time can enable less experienced ophthalmologists to learn the retinal laser photocoagulation approach in an intuitive manner during the telemedicine process, in contrast to traditional telemedicine.

### Limitations

However, there are certain limitations to the present telemedicine model. Further studies will be needed to compare this approach with the current standard care to determine whether visual acuity or safety outcomes differ. This study cannot determine whether these procedures sacrifice vision outcomes, nor can it rule out the potential for moderate common complications until several hundred participants have been evaluated. As such, a randomized clinical trial will be needed in the future to validate and expand on these findings. In addition, high-speed network construction and navigated laser devices are both very expensive, and policy solutions are necessary to improve access to these technologies. National financial support and the establishment of a national network are vital to the advancement of telemedicine. Our real-time telephotocoagulation approach has been shown to be safe, but studies of approaches to minimizing its costs are still required. The current telemedicine model may not be as necessary for countries like the United States that have an abundance of fundus specialists. It may be more feasible for developing countries such as China where network development is proceeding rapidly in many areas while obtaining sufficient medical resources remains challenging. The design of novel navigated laser devices that are less expensive and the development of other networks (including 5G, optical, satellite, and Starlink networks) may make our telemedicine approach more practical to implement. Other universal limitations to telemedicine include certain legal barriers and reimbursement, which were also relevant in the context of our laser photocoagulation for patients with DR.^4^ Efforts to overcome these barriers, which include liability concerns, credentialing at multiple clinics, and reimbursement policies, will be required to expand access to telehealth.

## Conclusions

In summary, we herein outlined a novel telemedicine paradigm that uses multiple tools and techniques, including a 5G high-speed network, navigated retinal laser photocoagulation, videoconferencing, and real-time monitoring to treat DR via laser photocoagulation therapy. This is the first such application of 5G technology to retinal laser real-time telephotocoagulation that we are aware of. Our telemedicine platform represents an approach to expanding the reach of medicine and improving patient-physician relationships, improving on traditional telemedicine by enhancing intervention adherence, and playing an educational role. This strategy may help overcome limitations to health care access and medical inequality in nations with limited health care resources. As digital technologies continue to evolve, we believe that current barriers to health care access will be overcome such that more individuals will be able to obtain quality care.

## Notes

Supplement. eMethods. Huzhou First People’s Hospital eFigure 1. Location of Peking Union Medical College Hospital and Huzhou First People’s hospital in China. eFigure 2. Patient journey flow chart of 5G tele-retinal laser photocoagulation
