# Supplementary material for: Application of 5G Technology to Conduct Real-Time Teleretinal Laser Photocoagulation for the Treatment of Diabetic Retinopathy
Source: JAMA Ophthalmol. 2021 Jul 8;139(9):1–9. doi: 10.1001/jamaophthalmol.2021.2312 (PMC8444028; doi:10.1001/jamaophthalmol.2021.2312)
Supplement: Supplement. — eMethods. Huzhou First People’s Hospital eFigure 1. Location of Peking Union Medical College Hospital and Huzhou First People’s hospital in China. eFigure 2. Patient journey flow chart of 5G tele-retinal laser photocoagulation [file jamaophthalmol-e212312-s001.pdf]

Supplemental Online Content

Chen H, Pan X, Yang J, et al. Application of 5G technology to conduct real-time tele–retinal laser photocoagulation for the treatment of diabetic retinopathy. *JAMA Ophthalmol*. Published online July 8, 2021.
doi:10.1001/jamaophthalmol.2021.2312

eMethods. Huzhou First People's Hospital
eFigure 1. Location of Peking Union Medical College Hospital and Huzhou First People's hospital in China**.**
eFigure 2. Patient journey flow chart of 5G tele-retinal laser photocoagulation

This supplemental material has been provided by the authors to give readers additional information about their work.

© 2021 Chen H et al. *JAMA Ophthalmology.*

eMethods. Huzhou First People’s Hospital

Huzhou is a prefecture-level city, with a population of 3.06 million residents. It is located in northern Zhejiang province in southeastern China and is 1200 km from Beijing (eFigure 1). Huzhou First People’s Hospital is a comprehensive level B tertiary hospital, but there are only two ophthalmologists on staff who can perform basic laser treatment. When patients in Huzhou require treatment by a more senior retinal specialist in PUMCH, they must travel a minimum of five hours by high-speed train and one hour by car or bus, in addition to the time required waiting for registration, making this journey impractical for most patients.

© 2021 Chen H et al. *JAMA Ophthalmology.*

eFigure 1. Location of Peking Union Medical College Hospital and Huzhou First People's hospital in China.

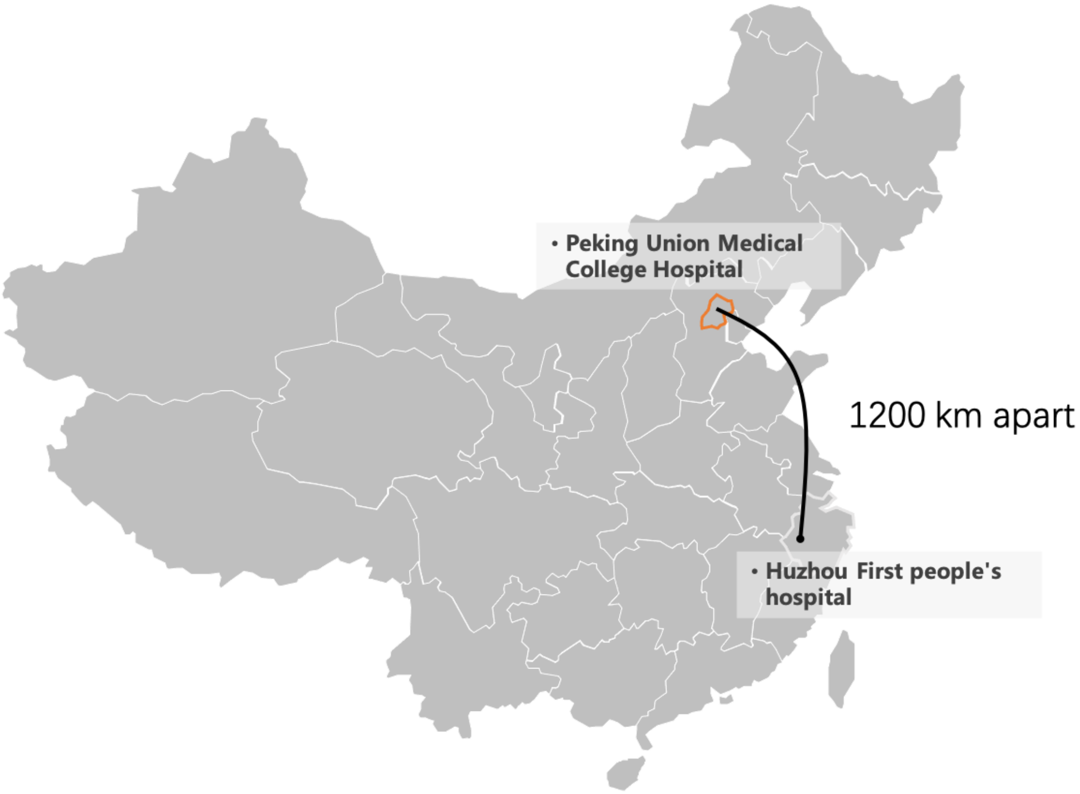

© 2021 Chen H et al. *JAMA Ophthalmology.*

eFigure 2. Patient journey flow chart of 5G tele-retinal laser photocoagulation

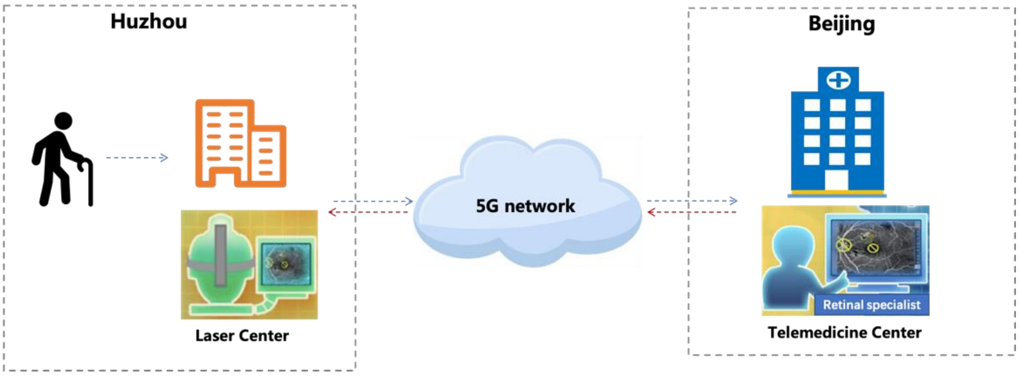

A DR patient in Huzhou visited Huzhou First People’s Hospital to undergo an ophthalmic examination. Their medical records were then sent to Beijing for review, and the retinal specialist located in Beijing was then able to discuss the patient’s treatment plan with the patient and their ophthalmologist in Huzhou using a video conference platform. The patient then underwent tele-retinal laser photocoagulation in the laser center of Huzhou First People’s Hospital, with the instrument being controlled remotely by the retinal specialist in Beijing using 5G network-based communications.

© 2021 Chen H et al. *JAMA Ophthalmology.*
